# Ambra1 Is an Essential Regulator of Autophagy and Apoptosis in SW620 Cells: Pro-Survival Role of Ambra1

**DOI:** 10.1371/journal.pone.0090151

**Published:** 2014-02-26

**Authors:** Wen Gu, Daiwei Wan, Qinyi Qian, Bin Yi, Zhilong He, Yilin Gu, Liang Wang, Songbing He

**Affiliations:** 1 Department of General Surgery, The First Affiliated Hospital of Soochow University, Suzhou, China; 2 Department of Hepatobiliary Surgery, The First Affiliated Hospital of Sun Yat-sen University, Guangzhou, China; 3 Institute of Health Science and Shanghai Institute of Immunology, Shanghai Institute for Biological Science, Chinese Academy of Science and Shanghai Jiao Tong University School of Medicine, Shanghai, China; 4 Department of Ultrasonography, Changshu No. 2 People’s Hospital, Changshu, China; 5 Department of Operating Rooms, The First Affiliated Hospital of SooChow University, Suzhou, China; 6 Department of Gastroenterology, The First Affiliated Hospital of SooChow University, Suzhou, China; 7 Washington University School of Medicine, St Louis, Missouri, United States of America; Istituto Nazionale per le Malattie Infettive, Italy

## Abstract

Recent research has revealed a role for Ambra1, an autophagy-related gene-related (ATG) protein, in the autophagic pro-survival response, and Ambra1 has been shown to regulate Beclin1 and Beclin1-dependent autophagy in embryonic stem cells. However, whether Ambra1 plays an important role in the autophagy pathway in colorectal cancer cells is unknown. In this study, we hypothesized that Ambra1 is an important regulator of autophagy and apoptosis in CRC cell lines. To test this hypothesis, we confirmed autophagic activity in serum-starved SW620 CRC cells by assessing endogenous microtubule-associated protein 1 light chain 3 (LC3) localization, the presence of autophagosomes (transmission electron microscopy) and LC3 protein levels (Western blotting). Ambra1 expression was detected by Western blot in SW620 cells treated with staurosporine or etoposide. Calpain and caspase inhibitors were employed to verify whether calpains and caspases were responsible for Ambra1 cleavage. To examine the role of Ambra1 in apoptosis, Ambra1 knockdown cells were treated with staurosporine and etoposide. Cell apoptosis and viability were measured by annexin-V and PI staining and MTT assays. We determined that serum deprivation-induced autophagy was associated with Ambra1 upregulation in colorectal cancer cell lines. Ambra1 expression decreased during staurosporine- or etoposide-induced apoptosis. Calpains and caspases may be responsible for Ambra1 degradation. When Ambra1 expression was reduced by siRNA, SW620 cells were more sensitive to staurosporine- or etoposide-induced apoptosis. In addition, starvation-induced autophagy decreased. Finally, Co-immunoprecipitation of Ambra1 and Beclin1 demonstrated that Ambra1 and Beclin1 interact in serum-starved or rapamycin-treated SW620 cells, suggesting that Ambra1 regulates autophagy in CRC cells by interacting with Beclin1. In conclusion, Ambra1 is a crucial regulator of autophagy and apoptosis in CRC cells that maintains the balance between autophagy and apoptosis.

## Introduction

Colorectal cancer (CRC) is one of the most common digestive cancers worldwide. Recently, combination therapy has improved the prognosis for CRC patients. However, the prognosis for advanced CRC with lymphatic metastasis remains poor because there are no effective therapies for this disease [1]. Chemotherapy resistance is a serious issue that is associated with poor prognosis and treatment problems [2], and autophagy may contribute to chemoresistance in CRC cells [3].

Autophagy is a highly conserved self-digestion process in eukaryotic cells that involves the degradation of old organelles and proteins to obtain energy. Increasing evidence suggests that the dysregulation of autophagic pathways is involved in various types of tumor clonal expansion and growth [4]–[8]. Autophagy serves a pro-survival function in CRC cell lines, and autophagy enhances the aggressiveness of CRC cells as well as their ability to adapt to apoptotic stimuli [9]. Additionally, autophagy rescues colorectal cancer cells from death in response to starvation or anti-tumor drugs [9], [10]. Autophagy is regulated by specific genes known as ATGs (autophagy-related genes). To date, more than 34 ATG genes have been identified in yeast. Ambra1 is a newly discovered ATG gene, and the Ambra1 protein is a crucial regulator of autophagy. Ambra1 interacts with Beclin1 through the target lipid kinase Vps34/PI3KC3 to assemble a class III PI3K complex, which positively regulates the formation of autophagosomes [11]. A dynamic interaction between Ambra1 and BCL-2 exists in mitochondria and potentially regulates Beclin1-dependent autophagy and apoptosis [12]. The function of Ambra1 in autophagy and apoptosis has been explored in vitro in embryonic stem cells and human fibroblast 2FTGH (2F) cells [13], but the role of Ambra1 in CRC cell lines has not been reported in the literature, and the role of this ATG protein in the autophagy and apoptosis pathways in CRC cell lines is unknown.

In this study, we used SW620 CRC cells to test the hypothesis that Ambra1 interacts with Beclin1 to promote autophagy and to inhibit apoptosis in CRC cell lines. We sought to determine whether autophagy occurs in SW620 CRC cells in response to apoptotic stimuli and whether Ambra1 regulates autophagy in SW620 cells by interacting with Beclin1. Our findings clearly suggest that Ambra1 functions at the intersection between autophagy and apoptosis. We found that Ambra1 interacts with Beclin1 to function as a pro-survival switch that inhibits apoptosis and induces autophagy, thereby preventing CRC cell death in response to apoptotic agents.

## Methods and Materials

### Cell and Culture

The human SW620 colorectal cell line was purchased from ATCC (American Type Culture Collection, Manassas, VA, USA) and cultured in Leibovitz’s L-15 medium (Invitrogen, Carlsbad, CA, USA) with 10% fetal bovine serum at 37°C in a 5% CO_2_ humidified atmosphere. Unless otherwise indicated, cells were treated with 5 µg/ml etoposide (Sigma-Aldrich, Shanghai, China) or 2 µM staurosporine (Sigma-Aldrich, Shanghai, China), both of which are apoptosis-inducing agents. All of the above drugs were solubilized in DMSO. Calpain inhibitor (CL) and caspase inhibitor (z-VAD-fmk) were used as previous described [14].

### RNA Interference

The following siRNA oligonucleotides corresponding to human Ambra1 cDNA were purchased from Genepharma (Shanghai, China): Ambra1 siRNA no. 1 5′-AGAACTGCAAGATCTACAA-3′ and Ambra1 siRNA no. 2 5′-GGCCCTATGGTACTAACAA-3′. The cells were transfected with 100 pmol Ambra1 siRNA using Lipofectamine RNAi max (Invitrogen, Carlsbad, CA, USA) in 6-well plates. The negative control siRNA was transfected under the same conditions. The transfection was repeated for two days to achieve a high efficiency. Approximately 24 h after the second transfection, 20×10^4^ cells/well were plated in 6-well plates and cultured with the indicated agents. Protein expression was measured by Western blot 48 h after transfection.

### Western Blot Analysis

The cells were washed twice with PBS containing 1 mmol/l phenylmethylsulfonylfluoride (PMSF). Next, the cells were scraped off the dishes and pelleted at 500×*g* for 10 min. The cells were subsequently lysed in cold lysis buffer (20 mmol/l Tris-HCl, 1 mmol/l EDTA, 150 mmol/l NaCl, 1 mmol/l EGTA, 1% Triton X-100, 2.5 mmol/l sodium pyrophosphate, 1 mmol/l β-glycerophosphate, 1 mmol/l Na3VO4, 1 µg/ml leupeptin and 1 mmol/l PMSF) and sonicated for 5 s. The lysates were clarified by centrifugation at 12000×*g* for 30 min at 4°C. Equal amounts of cell lysate were resolved by 8 or 15% SDS-PAGE. The membranes were blocked for 1 h in 5% powdered milk in TBST (10 mmol/l Tris-HCl, 150 mmol/l NaCl and 1% Tween-20). The membranes were subsequently immunoblotted using anti-Ambra1, anti-Beclin1, anti-LC3, anti-PARP, anti-Cathepsin D, anti-ATG5, anti-VPS34 and anti-β-actin antibodies (Cell Signaling Technology, Danvers, MA, USA). The PVDF membranes were incubated with a horseradish peroxide-conjugated anti-rabbit IgG antibody (Santa Cruz Biotechnology, CA, USA) for 1 h at room temperature. Immunoreactive bands were exposed to film (Kodak, Rochester, NY, USA). The band intensity was semi-quantified using BandScan software after scanning the blots (V300; EPSON, Tokyo, Japan).

### Real-time PCR

Real-time PCR was performed as previously described [14]. Each assay was performed in triplicate, and the average absorbance was calculated.

### Immunofluorescence Staining

The cells were fixed in 4% paraformaldehyde for 10 min and then washed with PBS for 5 min. The slides were immersed in 100 µg/ml digitonin for 15 min at room temperature and then washed. Subsequently, the cells were incubated with an anti-LC3 antibody (MBL, Nagoya, Japan) and washed 3 times. A FITC-conjugated anti-rabbit IgG antibody (MBL, Nagoya, Japan) was added to the cells, and the cells were washed after the incubation. Autophagosomes were examined using a fluorescence microscope (Nikon Eclipse TE200 microscope, Nikon, Inc., Melville, NY).

### Cell Viability Assays

Cells were plated at a density of 1×10^4^ cells/well in 96-well plates. The negative control cells were treated with 1% DMSO. Fifteen microliters of MTT was added to each well after 48 h, and the cells were incubated for an additional 4 h. Subsequently, 100 µl of the solubilization solution/stop mix was added according to the manufacturer’s instructions (Promega, Madison, WI, USA), and the plates were incubated for 60 min. The absorbance was measured at 570 nm and 630 nm using an ELISA reader. The actual counts were calculated by subtracting the absorbance at 570 nm from that at 630 nm. Each assay was performed in triplicate, and the average absorbance was calculated.

### Apoptosis Analysis

The Annexin-V-PE Apoptosis Detection kit (Invitrogen, Carlsbad, CA, USA) was used to measure apoptosis. Cells were washed with PBS and resuspended in 1X binding buffer at a concentration of 1×10^6^ cells/ml. Subsequently, 5 µl of annexin-V-PE and 5 µl of PI were added to 100 µl of the cell suspension, and the mixture was incubated for 15 min in the dark. After incubation, 400 µl of 1X binding buffer was added. The analyses were performed using a FACScan flow cytometer (Beckman Instruments, Fullerton, CA, USA).

### Electron Microscopy

SW620 cells were cultured in serum-free medium for 2, 4 or 8 h. The cells were fixed with 2% paraformaldehyde and 2% glutaraldehyde in 0.1 ml/L phosphate buffer (pH 7.4) followed by 1% osmium tetroxide. After dehydration, thin sections were stained with uranyl acetate and lead citrate and observed using a JEM 100 CX electron microscope (JEOL, Peabody, NY, USA).

### Co-immunoprecipitation Assays

The supernatants from the CRC cell lysates were incubated with anti-sera bound to protein A-sepharose beads (Amersham Bioscience) for 2 h at 4°C and washed extensively. To elute the bound proteins, 100 mM glycine-HCl (pH 2.5, precipitated with 5% trichloroacetic acid) was added, and the samples were washed with ice-cold acetone and resuspended in SDS sample buffer.

### Statistical Analysis

The statistical analyses were performed with SPSS. Statistical significance was determined using Student’s t-test. A p-value equal to or less than 0.05 was considered significant.

## Results

### Serum Deprivation-induced Autophagy is Associated with Ambra1 Upregulation in the SW620 CRC Cell Line

**Figure 1:**
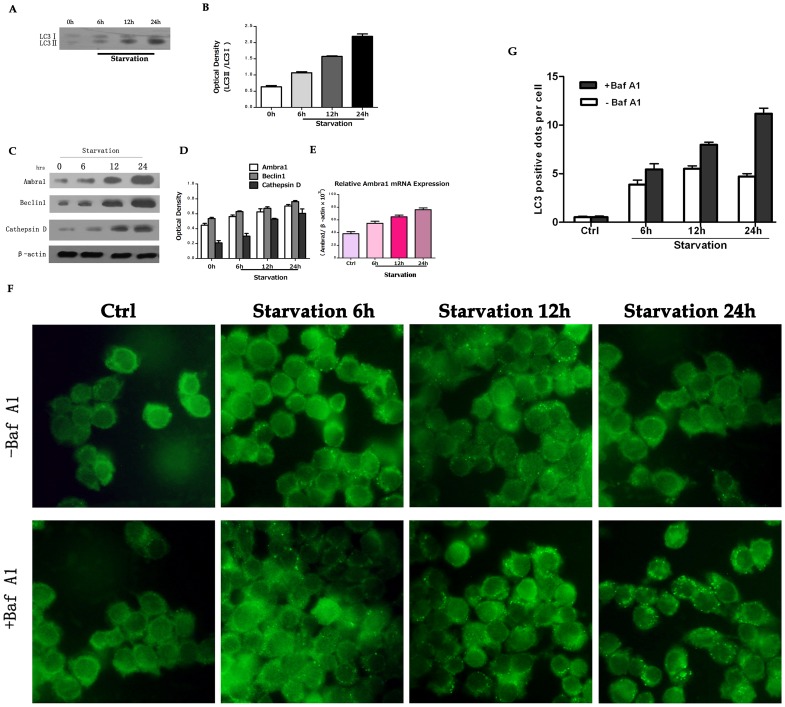
Autophagy is induced by starvation in SW620 cells. A: Western blot analysis of LC3 expression in SW620 cells. B: Quantitation of the optical density of LC3-II/LC3-I at various time points. C: Western blot of Ambra1, Beclin1 and cathepsin D expression after serum starvation at various time points. D: Quantitation of the optical density of Ambra1, cathepsin D and Beclin1. E: The relative Ambra1 expression after serum starvation for the indicated times as determined by real-time PCR. F: Immunofluorescent images of SW620 cells treated with or without 100 nM BafA1 for the indicated times. G: Quantification of autophagy in SW620 cells after serum starvation. The data are presented as the mean ± SD of three independent experiments (p<0.05).

We induced autophagy by starvation. The SW620 cells were cultured in serum-free medium for various time periods. LC3 is a homolog of the yeast autophagy-related protein Apg8p [15] and is used to mark the initiation of autophagy [16], [17]. The C-terminal fragment of LC3 is cleaved immediately after synthesis to yield a cytosolic form termed LC3-I. A subpopulation of LC3-I is further converted to the LC3-II autophagosome-associated form, which can be detected with the appropriate antibody. We used immunoblot analysis to measure the levels of LC3 and other ATG-related proteins in the SW620 starvation-induced autophagy model. As shown in Figure 1A, incubating SW620 cells in serum-free media in the presence of BafA1, a lysosome inhibitor, resulted in LC3-II accumulation. The LC3-II/LC3-I ratio increased after incubation with BafA1 in serum-free media. Interestingly, Ambra1 levels increased during autophagy as well as Beclin1 levels (Figure 1C). We measured the alterations in cathepsin D expression because autophagy is a constitutive process involving the activation of lysosomal enzymes and the subsequent degradation of substrates [18]. Cathepsin D expression was upregulated by serum starvation (Figure 1C). The quantification of the optical densities of Ambra1, Beclin1 and cathepsin D in serum-starved SW620 cells is presented in Figure 1D. We performed real-time PCR to distinguish whether Ambra1 upregulation was due to post-translational or transcriptional regulation, and we determined that Ambra1 mRNA expression was increased (Figure 1E). This finding suggested that the increased Ambra1 expression resulted from transcriptional regulation.

**Figure 2:**
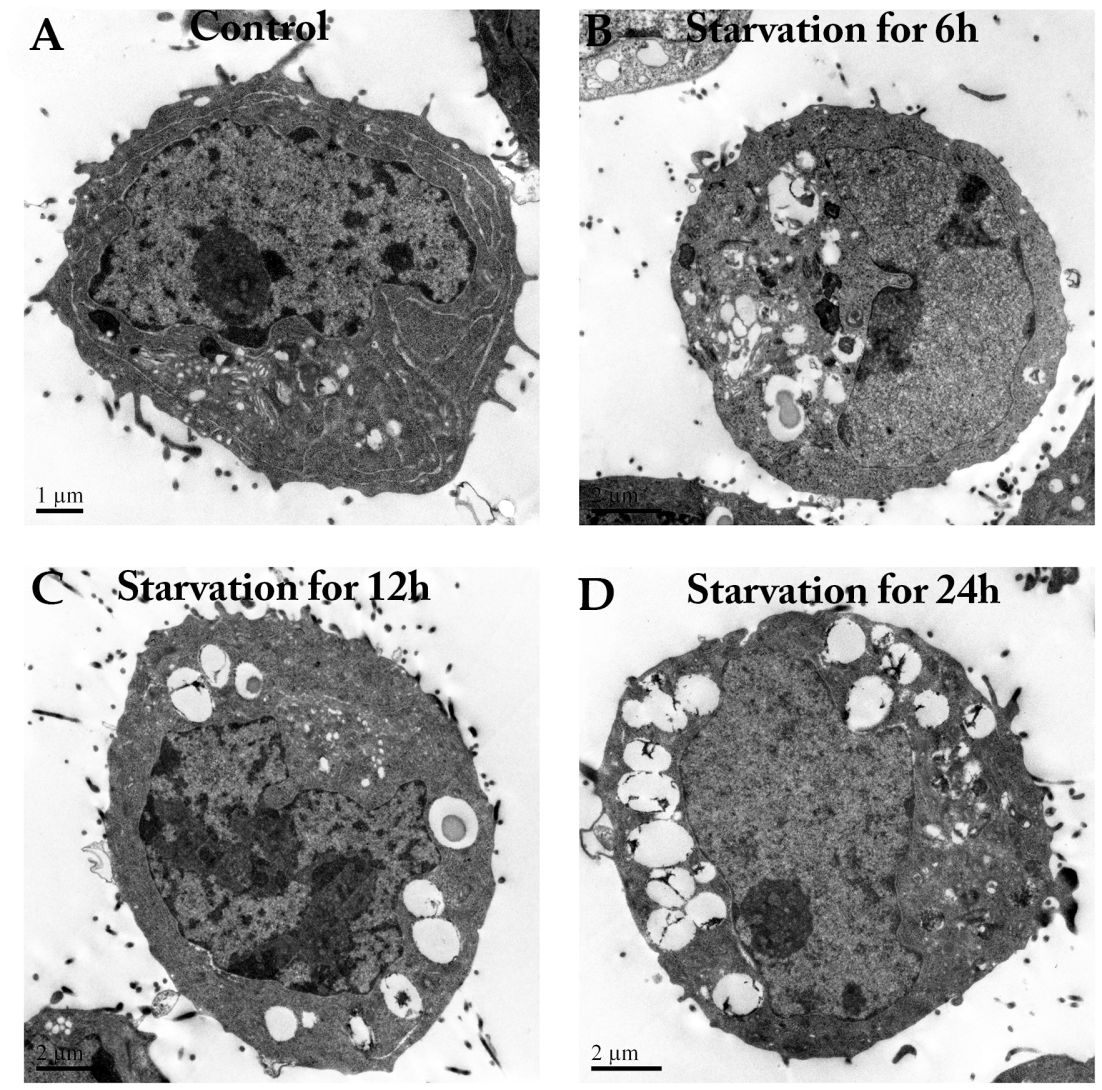
Representative electron micrographs of starvation-induced autophagy in SW620 cells at various time points. A: Control group. B: Starvation for 6 h. C: Starvation for 12 h. D: Starvation for 24 h.

To further confirm that the autophagosomes were induced by starvation, we examined serum-starved SW620 cells via fluorescence microscopy and transmission electron microscopy (TEM). An antibody against endogenous LC3 was used to detect autophagy by immunofluorescence. Endogenous LC3 was redistributed from a diffuse pattern to visible cytoplasmic puncta (Figure 1F). Figure 1G illustrates the average number of LC3 positive dots in SW620 cells. The TEM images confirmed the induction of autophagy at various time points as indicated by autophagosome visualization (Figure 2). These data demonstrated that autophagy was triggered by starvation and that Ambra1 was associated with autophagy.

### Ambra1 Expression Decreases during Staurosporine- or Etoposide-induced Apoptosis

**Figure 3:**
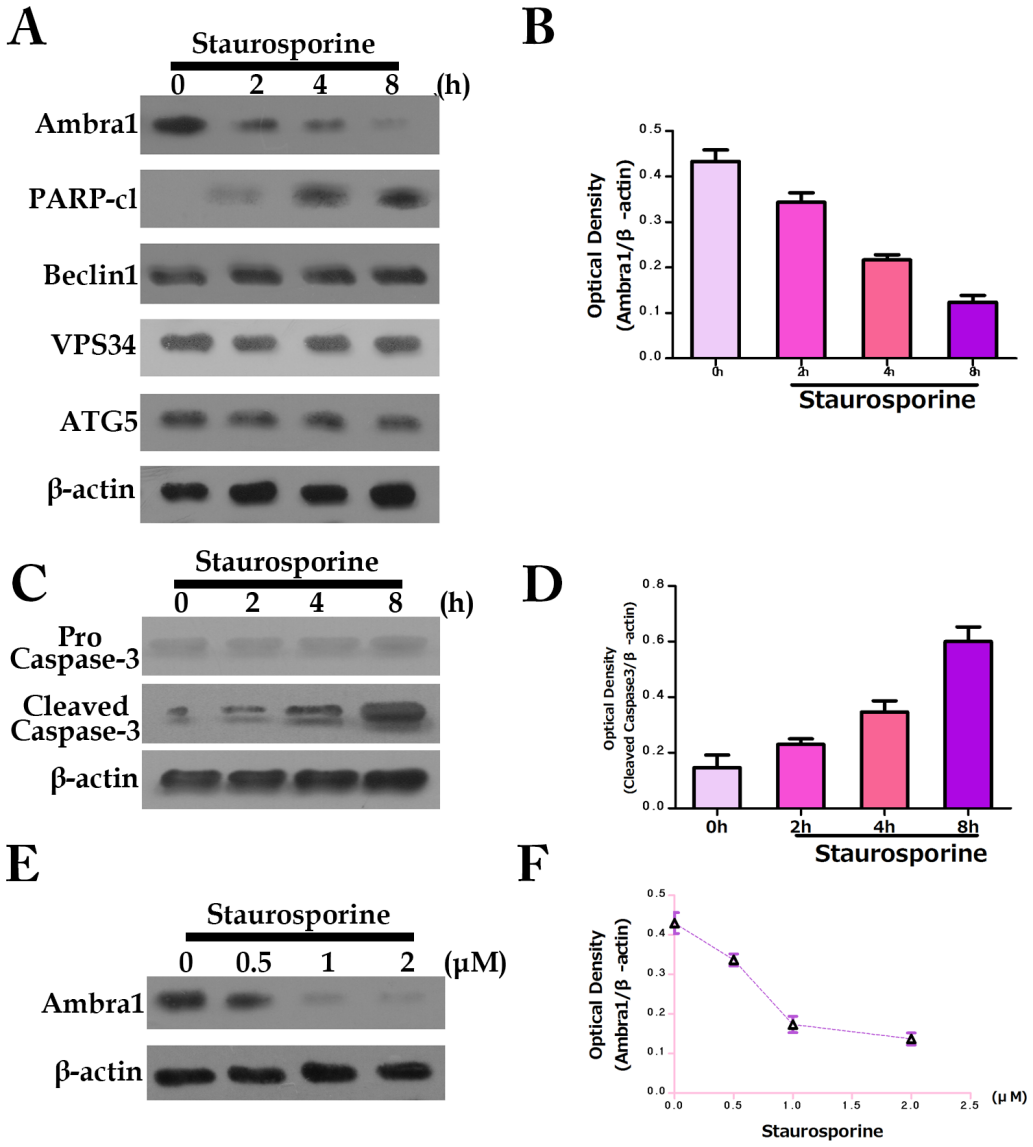
Ambra1 degradation during staurosporine-induced apoptosis. A: SW620 cells treated with 1 µM staurosporine. Ambra1, Beclin1, Vps34 and ATG5 expression as well as the appearance of cleaved PARP fragments were analyzed by Western blot at different time points. B: Normalized quantitation of the Ambra1 optical density in SW620 cells treated with staurosporine for the indicated times. C: SW620 cells treated with 1 µM staurosporine for the indicated times and analyzed by Western blot for pro-capsase-3 and cleaved caspase-3. D: Normalized quantitation of the cleaved caspase-3 optical density in SW620 cells treated with staurosporine for the indicated times. E: SW620 cells treated with various doses of staurosporine for 6 h. Ambra1 levels were measured by Western blot. F: Normalized quantitation of the Ambra1 optical density in SW620 cells treated with the indicated doses of staurosporine.

**Figure 4:**
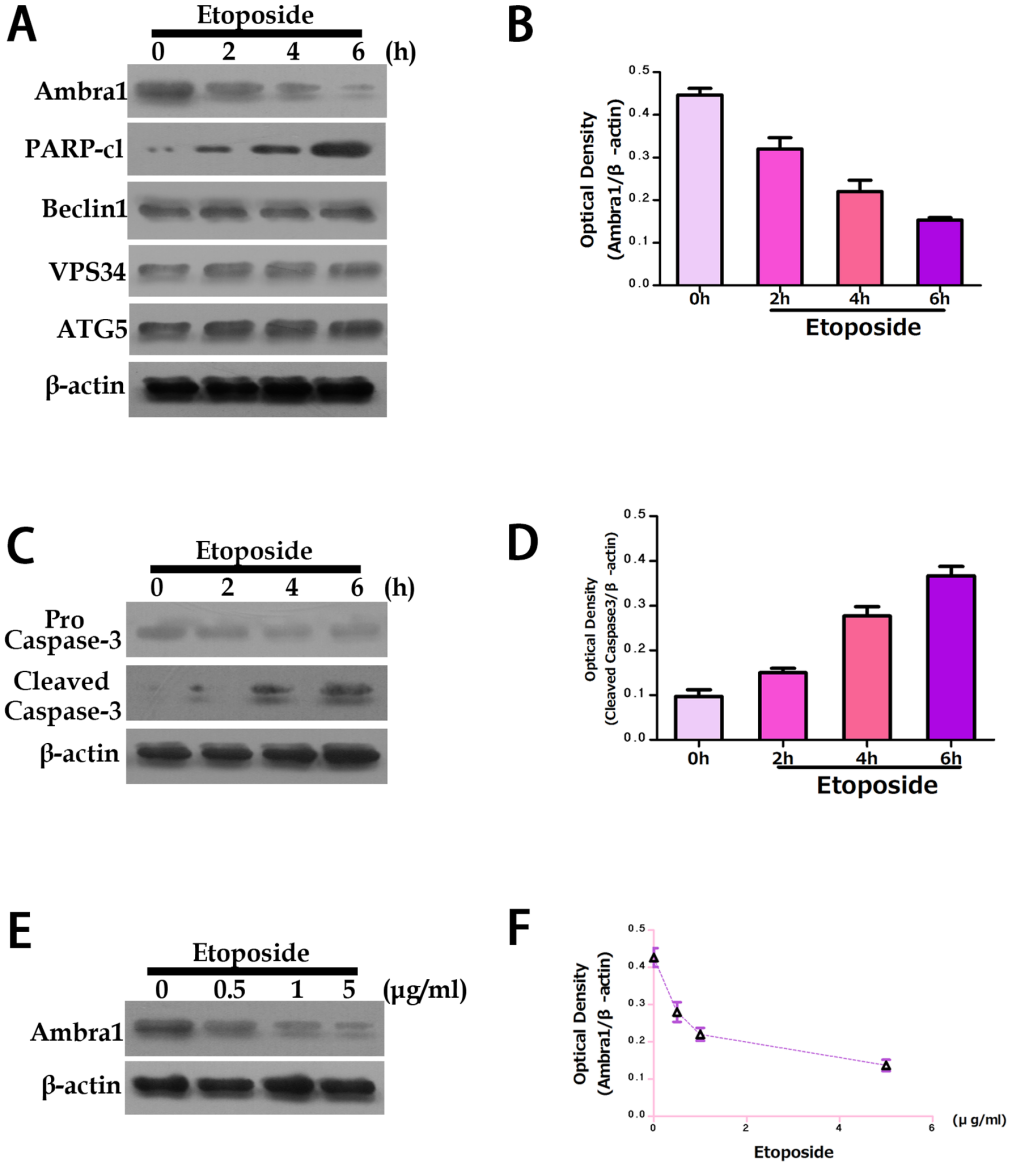
Ambra1 degradation during etoposide-induced apoptosis. A: SW620 cells treated with 1 µM staurosporine. Ambra1, Beclin1, Vps34 and ATG5 expression was measured by Western blot at various time points. B: Normalized quantitation of the Ambra1 optical density in SW620 cells treated with etoposide for the indicated times. C: SW620 cells treated with 1 µM staurosporine for the indicated times and analyzed by Western blot for pro-capsase-3 and cleaved caspase-3. D: Normalized quantitation of the cleaved caspase-3 optical density in SW620 cells treated with etoposide for the indicated times. E: SW620 cells treated with various doses of etoposide for 6 h. Ambra1 levels were measured by Western blot. F: Normalized quantitation of the Ambra1 optical density in SW620 cells treated with the indicated doses of staurosporine. The data are presented as the mean ± SD of three independent experiments (p<0.05).

Numerous pro-autophagic proteins are cleaved during apoptosis [19]. This cleavage may inhibit autophagy, thereby indirectly affecting cellular fate. Therefore, we analyzed Ambra1 expression in SW620 cells by Western blot after treatment with the apoptosis inducer staurosporine. We observed a notable decrease in Ambra1 protein expression after staurosporine treatment (Figure 3A). However, we did not observe a change in the levels of ATG5, Beclin1 or VPS34; decreased expression of these proteins has been reported during apoptosis [20], [21]. Caspase activation was detected by PARP cleavage concurrent with Ambra1 degradation. Furthermore, caspase-3 was cleaved during staurosporine-induced apoptosis (Figures 3C and D), indicating that Ambra1 downregulation occurred in parallel with caspase-3 activation. In addition, cells were treated with various doses of staurosporine to confirm the upregulation of Ambra1 (Figures 3E and F). To determine whether Ambra1 degradation and caspase-3 activation are common events, we analyzed Ambra1 and caspase 3 expression in etoposide-treated SW620 cells and observed a similar pattern of reduced Ambra1 expression and increased cleaved caspase-3 (Figures 4A–F).

**Figure 5:**
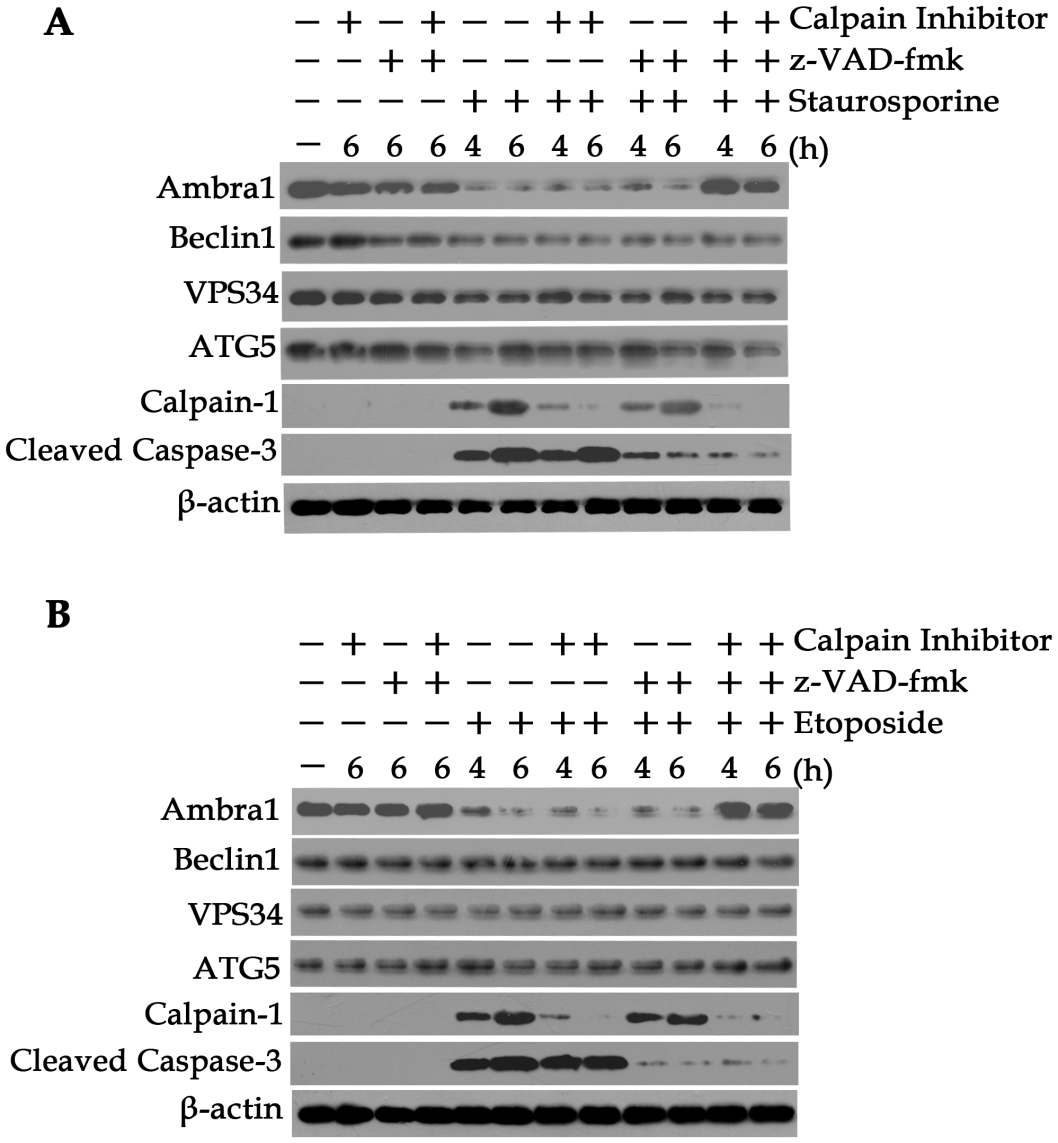
Calpains and caspases are responsible for Ambra1 cleavage. A: SW620 cells treated with 2 µM staurosporine for 6 h in the presence or absence of 20 µM calpain inhibitor (CL) and/or 10 µM caspase inhibitor (z-VAD-fmk). Ambra1 expression was evaluated by Western blot. B: SW620 cells treated with 1 µg/ml etoposide for 6 h in the presence or absence of 20 µM calpain inhibitor (CL) and/or 10 µM caspase inhibitor (z-VAD-fmk). Ambra1 expression was evaluated by Western blot. The data are presented as the mean ± SD of three independent experiments (p<0.05).

In a previous study, calpains and caspases were shown to be responsible for Ambra1 cleavage in fibrosarcoma cell lines [14]. To confirm whether Ambra1 is a target of these proteases during apoptosis in a CRC cell line, we treated SW620 cells with staurosporine in the presence of a calpain inhibitor (CL) and a caspase inhibitor (z-VAD-fmk). As shown in Figures 5A and B, staurosporine- and etoposide-induced Ambra1 cleavage was only inhibited when the cells were treated with the calpain and caspase inhibitors in combination, indicating that Ambra1 degradation during apoptosis may be mediated by both calpains and caspases. These results indicated that Ambra1 expression was downregulated and that calpains and caspases were responsible for Ambra1 cleavage during apoptosis in CRC cell lines.

### Serum Deprivation Induces Cell Death when Ambra1 is Inhibited

**Figure 6:**
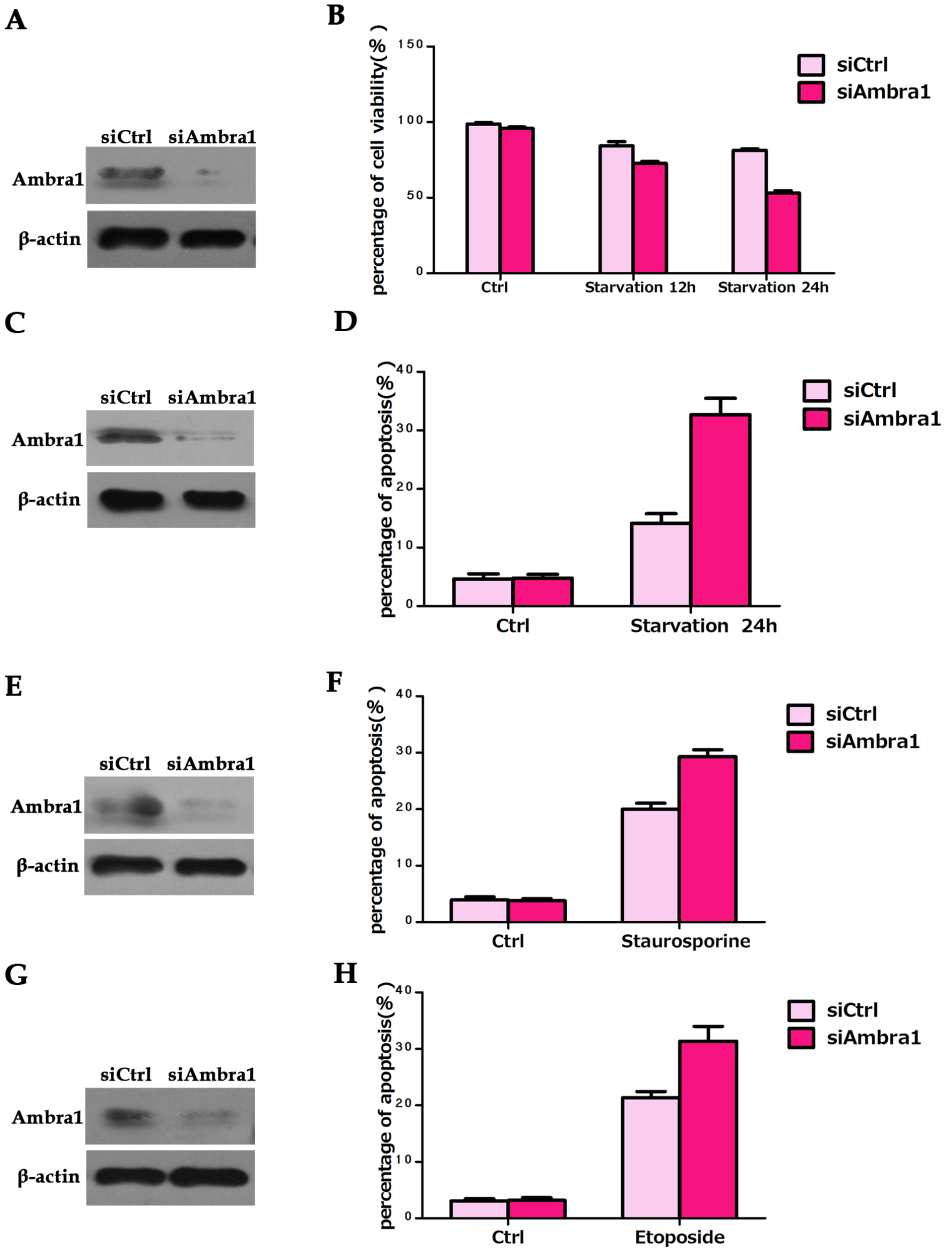
The pro-survival function of Ambra1 in SW620 cells. A: Western blot analysis of Ambra1 expression in SW620 cells after transient transfection with negative control or Ambra1-siRNA oligonucleotides. These cells were analyzed by MTT assay. B: SW620 cells cultured in serum-free medium for 12 or 24 h. Cell viability was determined by MTT assay. C: Western blot analysis of Ambra1 expression in SW620 cells after transient transfection with negative control and Ambra1-siRNA oligonucleotides. These cells were analyzed by flow cytometry. D: SW620 cells serum starved for 24 h. Cell death was measured by flow cytometry. E: Western blot analysis of Ambra1 expression in SW620 cells after transient transfection with negative control or Ambra1-siRNA oligonucleotides. These cells were assayed for staurosporine-induced cell death. F: SW620 cells treated with staurosporine (2 µM for 6 h). Cell death was measured by flow cytometry. G: Western blot analysis of Ambra1 expression in SW620 cells after transient transfection with negative control or Ambra1-siRNA oligonucleotides. These cells were assayed for etoposide-induced cell death. H: SW620 cells treated with etoposide (5 µg/ml for 24 h). Cell death was measured by flow cytometry. The data are presented as the mean ± SD of three independent experiments (p<0.05).

**Figure 7:**
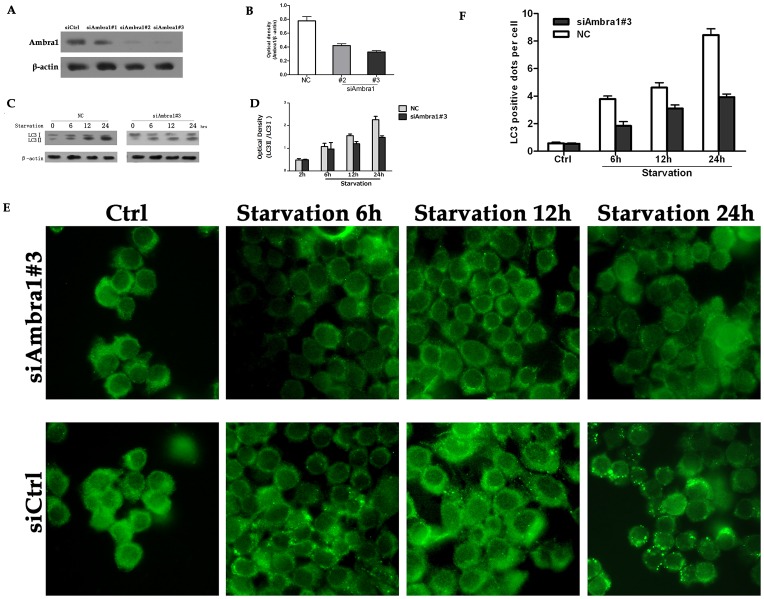
Ambra1 downregulation suppresses autophagy. A: Western blot analysis of Ambra1 expression in SW620 cells after transient transfection with negative control or Ambra1-siRNA oligonucleotides. B: Quantitation of the optical density of the Ambra1 bands after transfection. C and D: LC3 levels in negative control and si-Ambra1 serum-starved SW620 cells. E: Immunofluorescence images of SW620 cells treated with 100 nM BafA1. SW620 cells were transfected with negative control or Ambra1-siRNA oligonucleotides. F: The quantification of positive dots in the negative control and si-Ambra1 groups. The data are presented as the mean ± SD of three independent experiments (p<0.05).

Ambra1 is an anti-apoptotic ATG protein that promotes autophagy [14]. Therefore, we hypothesized that Ambra1 was a negative regulator of apoptosis in CRC cell lines. To verify this hypothesis, we knocked down Ambra1 expression in SW620 cells using siRNA (Figure 6A). The cell viability after Ambra1 knockdown was quantitated using an MTT assay. Serum deprivation (12 and 24 h) slightly decreased cell viability in all treatment groups. However, cell viability was markedly lower in the nutrient-starved Ambra1 siRNA group compared with the nutrient-starved negative control siRNA group (Figure 6B). Using the Ambra1 knockdown SW620 cells, we explored whether Ambra1 downregulation would result in increased cell death under stress (Figure 6C). In SW620 cells, the siCtrl groups were more resistant to stress-induced apoptosis than the siAmbra1 groups after 24 h of serum starvation (Figure 6D). Ambra1 expression was knocked down by siRNA to determine whether this rendered the cells more sensitive to apoptosis (Figure 6E). SW620 cells were stained with annexin-V and PI and analyzed by flow cytometry. Staurosporine treatment resulted in minimal cell death, whereas significant cell death was detected in the Ambra1 siRNA groups (Figure 6F). Similar results were obtained in Ambra1-siRNA cells treated with etoposide (Figures 6G and H). These results indicated that Ambra1 may be an anti-apoptotic factor in CRC cell lines. Ambra1 was inhibited to determine the function of Ambra1 in autophagy (Figures 7A and B). The siCtrl and siAmbra1 SW620 cells were cultured in serum-free medium in the presence of BafA1, and LC3-II expression was measured by Western blot. We observed LC3-II accumulation in the negative control and the Ambra1 siRNA groups (Figures 7C and D). Nevertheless, there was a greater decrease in the LC3-II/LC3-I ratio in the AMBRA siRNA group than in the negative control group. We dynamically monitored autophagosome formation by immunofluorescence in Ambra1 siRNA and negative control cells cultured in serum-free media. The results revealed a significant reduction in visible dots in the Ambra1 siRNA groups compared with the negative control groups (Figures 7E and F). These data suggested that Ambra1 was pro-autophagic in CRC cell lines.

### Ambra1 Triggers Autophagy by Binding to Beclin1

**Figure 8:**
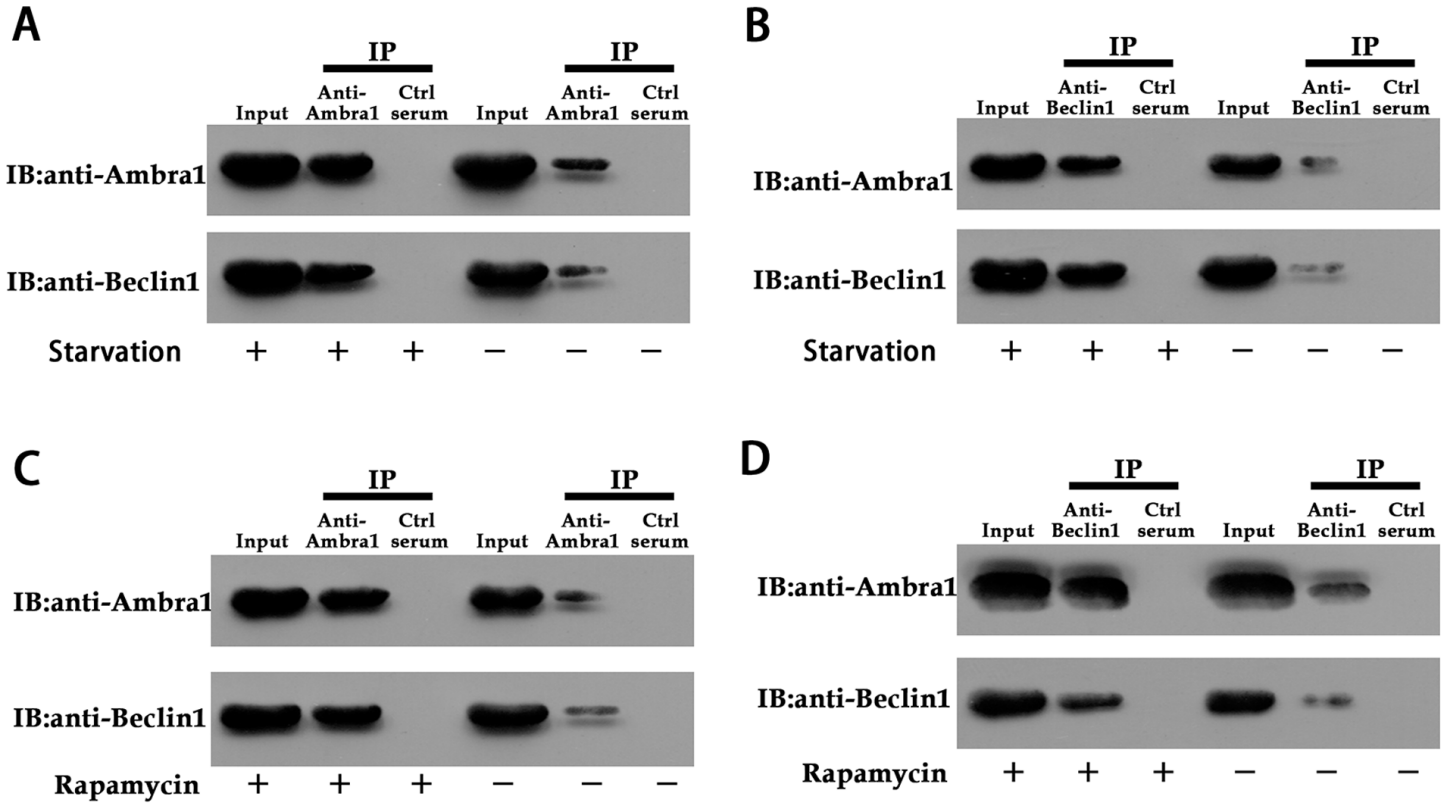
Ambra1 interacts with Beclin1 in SW620 cells. A and B: Co-immunoprecipitation assays with SW620 cell lysates. SW620 cells were serum-starved or not (control) for 12 h. Next, the cells were incubated with anti-Ambra1 antisera or control sera (A) or anti-Beclin1 antisera or control sera (B) covalently bound to protein A-sepharose beads. After extensive washes, the eluted proteins were separated and subjected to Beclin1 and Ambra1 immunoblotting. The procedure for (C) and (D) is similar to that for (A) and (B) with the exception that the cells were treated or not with rapamycin (100 nM for 12 h) instead of being serum-starved.

To understand how Ambra1 triggers autophagy, we investigated the mechanism by which Ambra1 affected autophagocyte formation. The Beclin1-Class III PI3K complex initiates autophagocyte formation; however, it has not been determined whether Ambra1 interacts with this complex in CRC cell lines. We determined whether endogenous Ambra1 interacted with endogenous Beclin1 upon the induction of autophagy in CRC cells. The interaction between endogenous Ambra1 and endogenous Beclin1 was confirmed by co-immunoprecipitation in SW620 cells. Using anti-Ambra1 or anti-Beclin1 antibodies to pull down the protein complexes, we observed that the Ambra1-Beclin1 interaction was more pronounced in autophagy-stimulated cells than in non-stimulated cells (Figures 8A and B). Similar results were observed in rapamycin-treated SW620 cells (Figures 8C and D). These findings confirmed that Ambra1 bound to Beclin1 during autophagy.

## Discussion

Ambra1 promotes autophagy in numerous cell types [22], but the role of Ambra1 in CRC remains unknown. In this study, we examined the role of Ambra1 in autophagy and apoptosis in SW620 colorectal cancer cells. Our novel findings suggested that Ambra1 not only promoted autophagy in this colorectal cancer cell line but also had the ability to control the switch between autophagy and apoptosis in these cells, thereby altering cellular fate. Additionally, our results indicated that Ambra1 induced autophagy in CRC cells by interacting with Beclin1.

Autophagy plays a role in determining cellular fate by degrading old organelles, recycling cellular components and responding to stressful cellular conditions [23]. The relationship between cancer and autophagy has been extensively studied in recent years [24], [25]. Our data are consistent with the work by Sato et al. that indicated that autophagy is activated in CRC in vitro and in vivo and that autophagy contributes to cancer cell survival in the cellular microenvironment [10]. In our study, immunofluorescent staining of the autophagosome marker LC3 revealed that autophagy was induced by serum-starvation in cancer cells. Autophagosome formation was confirmed by TEM.

Previous studies have indicated that Ambra1 regulates autophagy in multiple cell types, including neuronal [10] and 2F [25] cells. However, the role of Ambra1 in CRC cells has not been reported. Our study demonstrated that starvation induced Ambra1 expression and that Ambra1 knockdown suppressed autophagy in response to starvation in SW620 CRC cells. These results suggested that Ambra1 is crucial for starvation-induced autophagy in CRC cell lines, and are similar to results reported in other cancer cell types, suggesting a universal role for Ambra1 in autophagy in cancer.

In this study, we demonstrated that the Ambra1-Beclin1 autophagic pathway that has been described in other cell types is conserved in SW620 CRC cells. Beclin1 is a phylogenetically conserved protein that is essential for autophagy. Beclin1 associates with PI3KCIII/Vps34 and other co-factors, such as Ambra1, to initiate autophagy [22]. Beclin1 also acts as a tumor promoter in colon cancer [26], and a previous study suggested that Ambra1 regulates autophagy during murine embryogenesis by activating Beclin1 [13]. Our co-immunoprecipitation results demonstrated that Ambra1 and Beclin1 interacted in serum-starved or rapamycin-treated CRC cells, suggesting that Ambra1 regulates autophagy by interacting with Beclin1 to induce autophagosome formation. Although this interaction has been described in other cell types, the interaction between Ambra1 and Beclin1 during autophagy and apoptosis in CRC cells had not been previously reported. Greater knowledge of autophagy induction in these cell lines could contribute to a better understanding of the chemoresistance mechanisms in CRC.

An increasing number of factors have been identified that regulate both apoptosis and autophagy. For example, Bcl-2 is a well-known inhibitor of both autophagy and apoptosis [27], [28] that promotes CRC cell survival during nutrient-stressed conditions [29]. Beclin1, VPS34 and ATG5 positively regulate autophagy and negatively regulate apoptosis in Ba/F3 cells and the rat RGC line [20], [21], [30]. However, in contrast to previous studies of other cell types, we did not observe a significant reduction in Beclin1, VPS34 or ATG5 protein expression in staurosporine-induced apoptotic CRC cells. We did observe decreased Ambra1 expression. These results suggested that the exact mechanisms of autophagy in CRC cell lines could slightly vary from those reported in other cell types and that Ambra1 may be a critical regulator of autophagy in CRC cells. We confirmed that Ambra1 protein levels decreased in response to apoptotic stimuli by treating SW620 cells with etoposide, demonstrating that decreased Ambra1 expression is a common event during apoptosis in CRC cell lines. This pattern suggested that Ambra1 could be an important negative regulator of apoptosis in CRC. We confirmed in CRC cells the previous suggestion that calpains and caspases are involved in Ambra1 degradation [14]. These data strengthened the recent hypothesis that impairing autophagy results in cell death. It will be important to ascertain whether this phenomenon occurs in CRC cells, as it will provide additional therapeutic strategies.

Previous studies have reported that a functional deficiency in Ambra1 in mice results in developmental defects, embryonic death associated with impaired autophagy and increased apoptosis in vitro [31]. Therefore, we hypothesized that Ambra1 is involved in the apoptosis of CRC cells. SW620 cells were treated with staurosporine and etoposide, and Ambra1 expression was knocked down using siRNA. Our results demonstrated that the SW620 cells with decreased Ambra1 expression were more sensitive to apoptosis induced by starvation, staurosporine or etoposide and that downregulating Ambra1 expression significantly reduced cell viability under nutrient deprivation. These results suggested that Ambra1 plays a pro-survival role in colorectal cancer cell lines.

Our study confirmed that Ambra1 is an important factor in the pathways that regulate tumor cell survival in SW620 CRC cells. However, questions remain regarding the function of Ambra1 in the regulation of autophagy and apoptosis in these cells. We discovered one Ambra1-related autophagy pathway, but additional pathways may exist in CRC cells. Furthermore, the mechanism by which Ambra1 inhibits apoptosis has not been fully elucidated in these cell lines. Reports have suggested that Ambra1 and BCL-2 dynamically interact in the mitochondria to regulate apoptosis [12]. Moreover, apoptosis can regulate autophagy, and autophagy can inhibit apoptosis [32], [33]. However, the detailed mechanisms by which apoptosis and autophagy are regulated in CRC cells have not been fully elucidated. Future studies in animal models with colon-specific overexpression or knockdown of Ambra1 are necessary to confirm that Ambra1 plays a key role in regulating autophagy and apoptosis in CRC cells in vivo.

In conclusion, this study ascertained the role of Ambra1 as a regulator of autophagy and apoptosis in the SW620 CRC cell line. We demonstrated that Ambra1 is a key pro-survival factor in SW620 cells that promotes autophagy by binding to Beclin1 and inducing Beclin1-mediated autophagy. We determined that Ambra1 knockdown rendered SW620 CRC cells more susceptible to apoptosis, suggesting that Ambra1 may be an important negative regulator of apoptosis in these cells. Future studies are necessary to explore the detailed mechanisms that link the regulation of autophagy and apoptosis in these cancer cells. In addition, in vivo animal studies are essential for exploring the role of Ambra1 in the pro-survival pathways that contribute to CRC tumor growth, survival and chemoresistance.
